# Inhibitory Effects of Hydroethanolic Leaf Extracts of *Kalanchoe brasiliensis* and *Kalanchoe pinnata* (Crassulaceae) against Local Effects Induced by *Bothrops jararaca* Snake Venom

**DOI:** 10.1371/journal.pone.0168658

**Published:** 2016-12-29

**Authors:** Júlia Morais Fernandes, Juliana Félix-Silva, Lorena Medeiros da Cunha, Jacyra Antunes dos Santos Gomes, Emerson Michell da Silva Siqueira, Luisa Possamai Gimenes, Norberto Peporine Lopes, Luiz Alberto Lira Soares, Matheus de Freitas Fernandes-Pedrosa, Silvana Maria Zucolotto

**Affiliations:** 1 Grupo de Pesquisa em Produtos Naturais Bioativos (PNBio), Laboratório de Farmacognosia, Departamento de Farmácia, Universidade Federal do Rio Grande do Norte, (UFRN), Natal, Rio Grande do Norte, Brazil; 2 Laboratório de Tecnologia & Biotecnologia Farmacêutica (TecBioFar), Programa de Pós-graduação em Ciências Farmacêuticas (PPgCF), Universidade Federal do Rio Grande do Norte (UFRN), Natal, Rio Grande do Norte, Brazil; 3 NPPNS, Departamento de Física e Química, Faculdade de Ciências Farmacêuticas de Ribeirão Preto-SP, Brasil; 4 Laboratório de Farmacognosia, Universidade Federal de Pernambuco (UFPE), Recife, Pernambuco, Brazil; Universidade Federal do Rio de Janeiro, BRAZIL

## Abstract

The species *Kalanchoe brasiliensis* and *Kalanchoe pinnata*, both known popularly as “Saião,” are used interchangeably in traditional medicine for their antiophidic properties. Studies evaluating the anti-venom activity of these species are scarce. This study aims to characterize the chemical constituents and evaluate the inhibitory effects of hydroethanolic leaf extracts of *K*. *brasiliensis* and *K*. *pinnata* against local effects induced by *Bothrops jararaca* snake venom. Thin Layer Chromatography (TLC) and High Performance Liquid Chromatography coupled with Diode Array Detection and Electrospray Mass Spectrometry (HPLC-DAD-MS/MS) were performed for characterization of chemical markers of the extracts from these species. For antiophidic activity evaluation, *B*. *jararaca* venom-induced paw edema and skin hemorrhage in mice were evaluated. In both models, hydroethanolic extracts (125–500 mg/kg) were administered intraperitoneally in different protocols. Inhibition of phospholipase enzymatic activity of *B*. *jararaca* was evaluated. The HPLC-DAD-MS/MS chromatographic profile of extracts showed some particularities in the chemical profile of the two species. *K*. *brasileinsis* exhibited major peaks that have UV spectra similar to flavonoid glycosides derived from patuletin and eupafolin, while *K*. *pinnata* showed UV spectra similar to flavonoids glycosides derived from quercetin and kaempferol. Both extracts significantly reduced the hemorrhagic activity of *B*. *jararaca* venom in pre-treatment protocol, reaching about 40% of inhibition, while only *K*. *pinnata* was active in post-treatment protocol (about 30% of inhibition). In the antiedematogenic activity, only *K*. *pinnata* was active, inhibiting about 66% and 30% in pre and post-treatment protocols, respectively. Both extracts inhibited phospholipase activity; however, *K*. *pinnata* was more active. In conclusion, the results indicate the potential antiophidic activity of *Kalanchoe* species against local effects induced by *B*. *jararaca* snake venom, suggesting their potential use as a new source of bioactive molecules against bothropic venom.

## Introduction

The genus *Kalanchoe*, one of the most important genera of the family Crassulaceae used in folk medicine, contains approximately 125 species [1]. Most notable among these species are *Kalanchoe brasiliesis* Cambess, native to Brazil, and *Kalanchoe pinnata* (Lamk) Pers. (S1 Fig), native to Madagascar [1, 2]. These species are indistinctively known by the same common name in Brazil as "saião" and "coirama," and are widely used in traditional medicine for their anti-inflammatory properties [3]. Several popular reports have indicated that these species could be useful for treating snakebites [4, 5, 6].

The main feature that allows the distinction between the *K*. *brasiliensis* and *K*. *pinnata* species is the leaf aspect, as *K*. *brasiliensis* shows the subcrenate leaf margin, while *K*. *pinnata* has well-defined crenate leaf [7]. However, for non-specialized people, this distinction is very difficult, which justifies their interchangeably use in folk medicine. Additionally, it is important to mention that *K*. *pinnata* is included in the National List of Medicinal Plants of Interest to the Brazilian Public Health System (*RENISUS*), which is a report published by the Brazilian Health Ministry that includes 71 species of medicinal plants with the potential to generate pharmaceutical products of interest in the Brazilian public health system [8]. One of the aims of the present work was to obtain data on the safety and efficacy of *K*. *pinnata* and *K*. *brasiliensis*, with a view to including *K*. *brasiliensis* in RENISUS since it is a native Brazilian medicinal plant. In the literature, few reports about the chemical constituents and pharmacology of *K*. *brasiliensis* were found [9, 10, 11], while several chemical and pharmacological studies for *K*. *pinnata* were described [12, 13, 14, 15]. However, no study provides a comparative analysis between both species, which are morphologically similar and often used interchangeably by the population, and even known popularly by the same common name [3].

The chemical constituents of *K*. *brasiliensis* are mainly glycosylated flavonoids derived from aglycone patuletin [9], while for *K*. *pinnata* the constituents described are mainly steroids, bufadienolids, and glycosylated flavonoid derivatives of quercetin, kaempferol and luteolin aglycones [12, 16, 17, 18]. The main pharmacological studies of *K*. *brasiliensis* evaluated the anti-inflammatory and imunomodulatory activities, and showed promising results [9, 10, 19, 20]. In addition to evaluating anti-inflammatory and immunomodulatory activities, pharmacological studies of *K*. *pinnata* have evaluated other activities, including leishmanicidal and anti-diabetic activities [12, 14, 15, 21].

Snake envenoming is an important public health hazard in many regions, particularly in tropical and subtropical countries [22]. Global estimation indicates that, worldwide, there are more than 5 million snakebites, leading to 25,000–125,000 deaths [23, 24]. In Brazil, data from the Ministry of Health shows that there are more than 25,000 snakebites per year [25]. The high morbi-mortality rate still has a great impact on the population and on health-care systems, especially in rural areas of Africa, Asia, Oceania, and Latin America [22, 23]. For this reason, snakebite was included in the 2009 World Health Organization (WHO) list of Neglected Tropical Diseases (NTDs) and was classified as a major neglected disease of the 21st century [23].

More than 90% of the snakebites reported every year in Latin America are caused by *Bothrops* species [26]. Bothropic venom can induce a qualitatively similar pathophysiological picture, characterized by immediate and prominent local tissue damage (including hemorrhage, edema and myonecrosis), cardiovascular alterations (especially hemorrhage and hypovolemic shock), coagulation disorders (most frequently blood incoagulability), and renal alterations (which could evolve into acute kidney injury) [26, 27]. Snake venom are constituted by a complex mixture of pharmacologically active polypeptides and proteins, some with enzymatic activity, responsible for the local and systemic effects of envenomation. The principal toxins found in *Bothrops* venoms are snake venoms metalloproteases (SVMPs), phospholipases A_2_ (PLA_2_), snake venoms serine proteases (SVSPs), and hyaluronidases [27, 28, 29, 30].

The specific treatment for *Bothrops* envenomation consists of the earliest possible intravenous administration of antibothropic serum (ABS) or, in absence of this, the association of antibothropic-crotalic (ABCS) or antibothropic-antilachetic (ABLS) serum, in a hospital environment [27, 31]. However, the antivenom therapy has some limitations, such as poor effectiveness to treat local effects, difficult access in some regions, risk of immunological reactions, and high cost [32]. The use of steroidal or non-steroidal anti-inflammatory drugs is common for the relief of pain and some local symptoms [27, 31, 32]. If antivenom administration is initiated rapidly after envenomation, inhibition of systemic effects is usually achieved successfully; however, neutralization of local tissue damage is more difficult. The low efficacy against local effects, as well as the increased time between accident and treatment, is related to the temporary or permanent disability observed in many victims of snakebites. It is estimated that 400,000 people are left with permanent disabilities after snakebites [22, 23, 26]. In this scenario, the search for new complementary therapies to treat snakebites is relevant and medicinal plants could be highlighted as a rich source of natural inhibitors and pharmacologically active compounds [33, 34]. A significant number of plants have been evaluated for their potential inhibitory activity of *Bothrops* venoms [5, 33, 34, 35, 36, 37].

The aim of this study is to comparatively characterize the chemical constituents of *K*. *brasiliensis* and *K*. *pinnata* species, and evaluate their ability to inhibit local effects induced by *Bothrops jararaca* snake venom.

## Materials and Methods

### Plant material

*Kalanchoe brasiliensis* leaves were collected in Macaíba city, Rio Grande do Norte State, Brazil, at coordinates 5°51’30”S 35° 21' 14"W, in October of 2013. The botanical identification was done by Dr. Maria Iracema Bezerra Loyola and a voucher specimen (n° 5468) was deposited at the Herbarium of the Centro de Biociências of the Universidade Federal do Rio Grande do Norte, Brazil. *Kalanchoe pinnata* leaves were collected in Doutor Severiano city, in the same State, at coordinates 06° 05' 40" S 38° 22' 29" W, in September of 2012. The botanical identification was done by Dr. Rúbia Santos Fonseca and a voucher specimen (n° 57335) was deposited at the Herbarium Prisco Bezerra of the Universidade Federal do Ceará, Brazil.

The collection of the plant material was conducted under authorization of Brazilian Authorization and Biodiversity Information System (SISBIO) (process number 35017).

### Snake venom

The lyophilized *Bothrops jararaca* snake venom was commercially purchased from Sigma-Aldrich (St. Louis, MO, USA) (product number V5625). The scientific use of the material was approved by the Brazilian Access Authorization and Dispatch Component of Genetic Patrimony (CGEN) (Process 010844/2013-9). The venom was dissolved in phosphate-buffered saline (PBS) and the concentration was expressed in terms of proteins content that was quantified with the Bradford dye-binding assay, using albumin as a standard [38].

### Animals

*Swiss* albino mice (30–35 g, 6–8 weeks old), from both sexes, provided by the vivarium of the Health Sciences Center from Universidade Federal do Rio Grande do Norte were used. The mice were matched by sex and age in all procedures. The animals were maintained under standard environmental conditions and fed with food and water *ad libitum*. On the day of the experiment, the mice were placed in the experimental room for at least 1 h to allow acclimatization prior to use. At the end of the experiments, the mice were killed with an overdose of sodium thiopental (100 mg/kg, i.p.). All the procedures involving mice were done attempting to preserve animal health, body condition and well-being, in agreement with the recommendations of the National Council for the Control of Animal Experimentation of Brazil (CONCEA) and the International Guiding Principles for Biomedical Research Involving Animals of the Council of International Organizations of Medical Sciences (CIOMS). The experimental protocols were approved by the Ethics Committee on Animal Use of the Universidade Federal do Rio Grande do Norte (protocol n° 014/2013).). Every attempt was made to minimize the number of animals used and their suffering. There was no unexpected death during the entire set of experiments.

### Hydroethanolic leaf extracts of *K*. *brasiliensis* and *K*. *pinnata* preparation

*K*. *brasiliensis* (3 kg) and *K*. *pinnata* (1.6 kg) fresh leaves were extensively washed and extracted with ethanol: water (1:1, v/v) by turbo extraction for 5 minutes at a plant: solvent proportion of 1:1 (w/v), in an industrial blender. The hydroethanolic extracts (HE) were filtered and concentrated by rotaevaporator (model V-700, Buchi). One part of each HE was freeze-dried for HPLC analysis and biological activities, while another part was used for TLC analysis. The freeze-dried HEs were dissolved in PBS containing 5% castor oil (Sigma-Aldrich, St. Louis, MO, USA) for the biological assays.

### Thin Layer Chromatography (TLC) profile of hydroethanolic extracts

Thin Layer Chromatography (TLC) was carried out on silica gel F_254_ (Merck, Darmstadt, Germany) using two different mobile phases: (1) ethyl acetate: formic acid: methanol: water (10:0.5:0.6:0.2 v/v/v/v) and (2) toluene: ethyl acetate: formic acid (5:5:0.5 v/v/v). For this analysis, the extracts were fractionated by liquid-liquid partition with solvents of increasing polarity in order to obtain the dichloromethane (CH_2_Cl_2_), ethyl acetate (EtAcO), and *n*-butanol (BuOH) fractions. After development, the plates were dried and the components observed under UV light (254 and 365 nm). The plates were sprayed with NP reagent (1% diphenylboryloxyethylamine in methanol; Sigma^®^) and visualized under UV-365 nm. The retention factors (*Rf*), color, and behavior of the spots were compared with chromatographic profiles of reference substances reported in the literature [39].

### High Performance Liquid Chromatography (HPLC) profile of hydroethanolic extracts

The hydroethanolic extracts of *K*. *brasiliensis* and *K*. *pinnata* leaves were analyzed by HPLC-DAD-MS and HPLC-DAD-MS/MS in order to chemically characterize the two species. The liquid chromatography (LC) system used was a Shimadzu Model LC-20AD, with DAD detector SPD-M20A coupled to a mass spectrometer ESI-IT (AmaZon SL, Bruker Daltonicx, Billerica, MA, USA). The analyses (MS, MS^2^ and MS^3^) were obtained in positive ion mode and nitrogen was used as the nebulizer gas at a pressure of 50 psi and a flow rate of 7 l/min for dry gas. The capillary voltage and temperature were set at 3500 V and 220°C, respectively, and helium was used as the collision gas. A Phenomenex RP-18 column (250 x 4.6 mm, 5 μm particle size) was used. The eluents were: (A) acetic acid 0.3% and (B) acetonitrile. The following gradient (v/v) was applied: 7–15% B, 0–5 min; 15–24% B, 5–35 min; 24–25% B, 35–43 min; 25–30% B, 43–60 min; 60 minutes total analysis time. Flow elution was 0.7 mL/min, and 20 μL of each sample was injected. The UV-DAD detector was programmed to wavelength 200–800 nm and the chromatograms were plotted at 340 nm. The lyophilized HE of *K*. *brasiliensis* or *K*. *pinnata* was resuspended in methanol: water, 1:1 (v/v) and the final concentration was 2 mg/mL. HPLC-grade acetonitrile purchased from Merck (Brazil) was used and acetic acid was provided by Vetec (Brazil). Water was purified with a Milli-Q system (Millipore). The samples and solvents were filtrated through a membrane (pore-size 0.45 μm) and degassed. The analyses were performed in triplicate.

### Inhibition of phospholipase A_2_ (PLA_2_) activity

The PLA_2_ activity of *B*. *jararaca* venom was determined turbidimetrically in 96-well microplates using an egg yolk suspension (1:3 v/v, egg yolk: PBS) as substrate, as previously described in literature, with some modifications [40]. For the assay, a 5% (v/v) egg yolk suspension was prepared in 50 mM Tris-HCl pH 7.4 containing 200 mM NaCl and 5 mM CaCl_2_. In each well 100 μL of the suspension was mixed with 100 μL of a solution containing 2.5 μg of venom pre-incubated for 30 min at 37°C alone or with different concentrations of HE of *K*. *brasiliensis* or *K*. *pinnata* (1:1–1:100 venom: HE, w/w) in PBS. After 30 min, the absorbance was read at 925 nm, using a microplate reader (Epoch-BioTek, Winooski, VT, USA). Blanks for each concentration were prepared in the same way, except for replacing the substrate by an equal volume of buffer. The PLA_2_ activity was calculated based on the decrease in turbidity compared to the control in which only substrate was incubated (absence of venom and/or extracts).

### Inhibition of local hemorrhagic activity

In the pre-treatment protocol, the hemorrhagic activity of *B*. *jararaca* venom was performed using the *in vivo* model of local hemorrhage, as previously described in the literature, with a few modifications [41, 42]. Groups of 5 animals were treated with different doses of HE of *K*. *brasiliensis* or *K*. *pinnata* (125, 250, and 500 mg/kg, in a volume of 10 mL/kg of body weight) by intraperitoneal (i.p.) route. After 60 min, the animals received a subcutaneous (s.c.) injection of 20 μg of venom (100 μL) in the ventral region of the abdomen. After 3 h, the animals were sacrificed and had the inner surface of the skin exposed. After photodocumentation of the hemorrhagic halos produced, the hemorrhagic skin was removed and weighed on an analytical balance. The hemorrhage was expressed as the mass of hemorrhagic halo formed after subcutaneous injection of venom in grams.

In another set of experiments, the inhibitory activity of both extracts was assessed in a post-envenomation protocol, as previously described in the literature [40]. For this purpose, group of 5 animals received a s.c. injection of 20 μg of venom (100 μL) in the ventral region of the abdomen, as described above. After 5 min of the envenomation, animals were treated with *K*. *brasiliensis* or *K*. *pinnata* HE (500 mg/kg, i.p.) and then euthanize 3 h post-venom. The ventral skin was subsequently removed and photographed, after which the hemorrhagic halo was excised, fragmented and homogenized with 1 mL of Drabkin’s solution for hemoglobin extraction. After incubation for 48 h at 8°C, the samples were centrifuged at 16,000 g for 30 min at 4°C. Then, 100 μL of the supernatant was read at 540 nm with a microplate reader (Epoch-BioTek, Winooski, VT, USA), using Drabkin’s solution as blank. The hemoglobin content of the excised hemorrhagic halo was calculated from a standard curve of hemoglobin. The hemorrhage was expressed as the hemoglobin content (mg/mL) extracted from hemorrhagic halos after subcutaneous injection of venom [35, 41].

### Inhibition of edematogenic activity

In the pre-treatment protocol, the edematogenic activity of *B*. *jararaca* venom was performed using the *in vivo* model of paw edema, as previously described in the literature with minor modifications [35]. Groups of 5 animals were treated with different doses of HE of *K*. *brasiliensis* and *K*. *pinnata* (125, 250, and 500 mg/kg, in a volume of 10 mL/kg of body weight) by i.p. route. An additional group consisted in the i.p. administration of dexamethasone (5 mg/kg), as an anti-inflammatory standard group. After 60 min, the animals received an intraplantar (i.pl.) injection of 3 μg of venom (in 50 μL of PBS) in the right hind paw. The venom dose was chosen based in pilot assays, where the selected dose induced significant paw edema without produce paw hemorrhage. The individual right hind paw thickness was further measured at 30, 60, 90, and 120 min after venom injection with a digital caliper (Digimess, São Paulo, SP, Brazil). Edema was expressed as the percentage difference between the thickness of the paw after and before venom injection, at each time-point. At the end of the assay, the animals were sacrificed and their right hind paws were removed and analyzed for myeloperoxidase (MPO) activity, as previously described in the literature, with a few modifications [35, 43]. Tissues were homogenated in 0.5% hexadecyltrimethylammonium bromide buffer (1 mL of buffer for each 50 mg of tissue), sonicated in ice bath for 3 min, submitted to 3 cycles of freeze-thaw and, finally, sonicated for 3 min once more for MPO enzyme extraction. The supernatant obtained after centrifugation at 10,000 rpm for 10 min at 4°C was used for determination of MPO activity. Thus, 50 μL of supernatant were mixed with 200 μL of 50 mM potassium phosphate pH 6.0 containing 0.0005% hydrogen peroxide and 0.167 mg/mL *o*-dianisidine. The MPO activity was colorimetrically determined using a microplate reader (Epoch-BioTek, Winooski, VT, USA), analyzing changes in absorbance at 460 nm for 5 min. The left hind paws (without treatment) from the venom control group were used as control (basal values).

In another set of experiments, the inhibitory activity of both extracts was assessed in a post-envenomation protocol, as previously described in the literature [40]. Group of 5 animals received an i.pl. injection of 3 μg of venom (in 50 μL of PBS) in the right hind paw, as described above. After 5 min of the envenomation, animals were treated with 500 mg/kg of *K*. *brasiliensis* or *K*. *pinnata*, or 5 mg/kg of dexamethasone, intraperitoneally. Edema determination and MPO quantification was performed as described above. The time, doses and rout was chose according to pilot assays.

### Statistical analysis

Numerical results are presented as mean ±standard error of mean (SEM). One-way analysis of variance (ANOVA) followed by Tukey post-hoc test and two-way ANOVA followed by Bonferroni test were used for statistical comparisons in conjunction with GraphPad Prism version 5.00 (San Diego, CA, USA). Values of p<0.05 were considered significant.

## Results and Discussion

### TLC profile of hydroethanolic extracts

For phytochemical analysis by TLC, the extracts were fractionated by liquid-liquid partition to obtain fractions with different polarities, thus facilitating the chromatographic analysis of the compounds.

The analyses by TLC showed a great variety of phenolic compounds, with differences between HEs and fractions of *K*. *brasiliensis* and *K*. *pinnata* (S2 Fig). The orange colors when revealed with NP Reagent and observed under UV-365 nm of the *K*. *brasiliensis* compounds indicate the presence of flavonoids, probably with a patuletin skeleton. The yellow colors of the *K*. *pinnata* compounds indicate the presence of flavonoid compounds, probably with a quercetin skeleton [39].

### HPLC-DAD-MS and HPLC-DAD-MS/MS profile of hydroethanolic extracts

**Fig 1:**
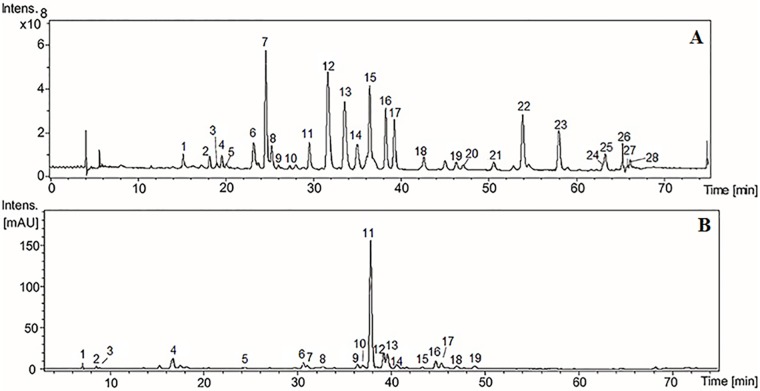
HPLC-DAD-MS chromatograms of *K*. *brasiliensis* (A) and *K*. *pinnata* (B) HE extracts. The extracts were chromatographed on a Phenomenex RP-18 column (250 x 4.6 mm, 5 um) and the column was eluted with a gradient of acetonitrile (ACN) in 3% acetic acid at a flow rate of 0.7 mL/min. The elution profile was monitored at 340 nm. Peaks 6–8, 11–17, 22 and 23 were considered to be major peaks in panel A, and peaks 4 and 11–13 were considered to be major peaks in panel B.

Chromatographic fingerprints obtained by HPLC-DAD-MS and HPLC-DAD-MS/MS of HE from *K*. *brasiliensis* and *K*. *pinnata* are depicted in Fig 1. It is possible to observe that *K*. *pinnata* presents a simpler chromatographic profile when compared with *K*. *brasileinsis* as well as observed in TLC analysis.

The chromatography HPLC-DAD profile of HE from *K*. *brasiliensis* (Fig 1A) exhibits at least ten major peaks, most of which have UV spectra similar to glycosylated flavonoid derivatives from patuletin, such as patuletin 3*-O*-glycoside (261 and 270 nm II band, and 355 nm I band) [44]. The isolation and characterization of patuletin derivatives has been described [9]. We have recently identified a patuletin glycoside of *K*. *brasiliensis* as patuletin 3-*O*-α-L-rhamnopyranosyl-7-*O*-α-L-rhamnopyranoside [45]. In the Fig 1A, this isolated compound was identified as the peak seven (7) due to comparing its fragmentation profile and standard analysis.

In the chromatogram of HE from *K*. *pinnata* (Fig 1B) was observed at least four major peaks at UV 340 nm. Most had UV spectra similar to glycosylated flavonoids derivatives of quercetin, as quercitrin (256 and 265 nm II band, and 355 nm I band) [44]. Isolation of glycosides derived from quercitin and kaempferol has been previously described in *K*. *pinnata* leaves [13, 14, 46].

In the hydroethanolic leaves extracts of the both species, the major compounds identified by HPLC-DAD-MS and HPLC-DAD-MS/MS correspond to flavonoids-*O*-glycosides (S1 and S2 Tables). The aglycones found were the flavon eupafolin and the flavonols patuletin, quercetin and kaempferol, the latter being identified only in the leaves of *K*. *pinnata*. The conjugates sugars with these structures are the hexoses (as glucose), pentose (as arabinose) and deoxy-hexoses (as rhamnose). The compounds were identified with base in the UV datas, mass spectra of precursor ions and ion product in mode MS^1^, MS^2^ and MS^3^.

The MS^3^ allowed us to isolate the aglycone ion at *m/z* at 333 (relative to the patuletin structure) and the complete scheme of fragmentation pathways were descried at supplementary material (S1 File). As expected the fragmentation reactions starts with a methyl group radicalar elimination well defined by computational methods [47, 48]. The classical Retro-Diels-Alder reactions for flavonoids [49] were not observed in high intensity (lower than 10% of total ions) by predominance of the radical rearmament that afford the keys fragment ions. The gas phase chemistry are fully in agreement with the proposed aglycone structure. S1 and S2 Tables showed the identified flavonoids-*O*-glycosides and MS^3^ analysis for all aglycones *m/z* at 333 confirm the same fragmentation pathway, confirming the patuletin core. For eupafolin it was expect the same initial radicalar elimination and MS^3^ spectra of the aglycone ion (*m/z* at 317) confirm the hypothesis (see scheme 2 in supplementary material–S1 File). The fragmentation pathway as in agreement with the eupafolin analogue and the only new reaction was a •H radicalar elimination which occur after a CO elimination. The occurrence of this elimination is inherent in open-shell or closed-shell formation, such as an anion radical or an anion previously described for isoflavonoids supported by computational methods [48]. The MS3 analysis relative to the aglycones quercetin and kaempferol are in agreement with previous published fragmentation data.

### Antiophidic activity evaluation of hydroethanolic extract

Studies that evaluate the antiophidic activity of *Kalanchoe* species are very scarce in the literature. To date, only two studies with *K*. *brasiliensis* were found showing its inhibitory potential against *B*. *alternatus* and *B*. *jararaca* snake venom-induced edema and skin hemorrhage [5, 11]. However, no studies have compared the abilities of *K*. *brasiliensis* and *K*. *pinnata* to protect against the local effects of *B*. *jararaca* venom, which is relevant to be noted since both vegetal species are interchangeably used in folk medicine as antiophidic, mainly due to the great morphological similarity of species [7]. So, in view of this, in the present paper, the inhibitory action of both plant species upon local effects resulting from envenoming was evaluated.

Snake venom phospholipases A_2_ (PLA_2_s) are a class of calcium-dependent enzymes and represent a superfamily of lipolytic enzymes which specifically catalyze the hydrolysis of the ester bond at the sn-2 position of glycerophospholipids, resulting in the generation of fatty acids (arachidonate) and lysophospholipids [50, 51]. In addition to their catalytic function, PLA_2_s show pharmacological activities that may be dependent or independent of catalytic property. Hemorrhagic, myotoxic, hemolytic, edematogenic, and neurotoxic activities has been attributed to PLA_2_, as a result, these enzymes have been implicated in many of the toxic and pharmacologic effects observed in snake envenomation [51, 52, 53].

**Fig 2:**
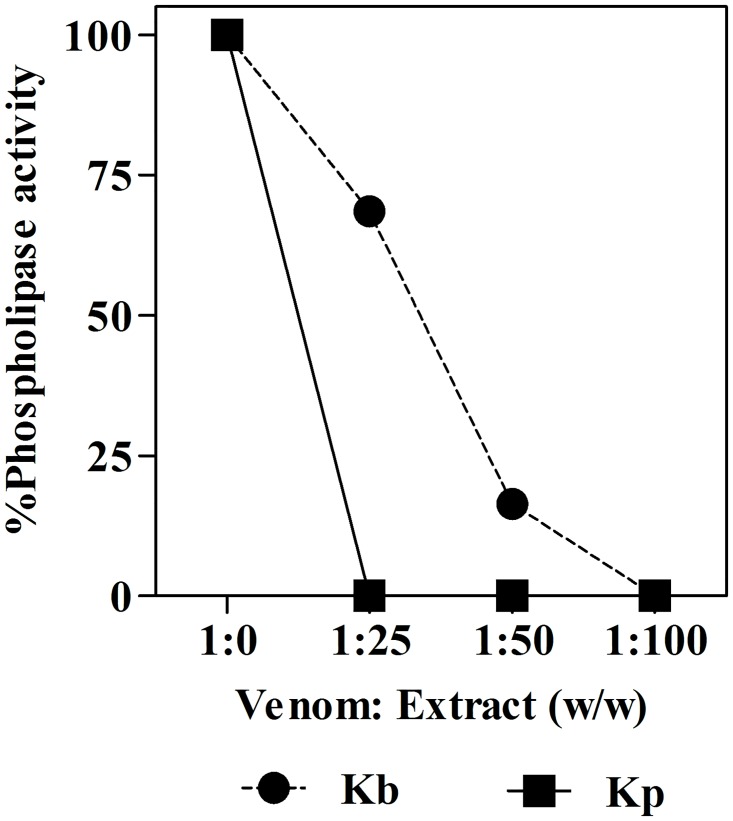
Inhibition of PLA_2_ activity of *B*. *jararaca* venom by a hydroethanolic extract from *K*. *brasiliensis* (Kb) (A) and *K*. *pinnata* (Kp) (B). The points are the mean ± SEM (n = 3). Note that the error bars are smaller than the symbols.

The results reveal that both extracts were able to inhibit the phospholipase activity induced by *B*. *jararaca* venom (Fig 2). However, the *K*. *pinnata* HE extract was more effective since at a venom: extract ratio of 1:25 (w/w) the PLA_2_ activity was completely inhibited, while *K*. *brasiliensis* completely inhibited the PLA_2_ activity only at a ratio four times greater (Table 1). These results suggest that both extracts can contained compounds capable of inhibiting PLA_2_ activity, with the *K*. *pinnata* extract being more potent.

**Table 1:** Inhibition of the enzymatic and biological activities of *B*. *jararaca* venom by hydroethanolic extracts of *K*. *brasiliensis* and *K*. *pinnata*.

Activity	Inhibition (%)	
	*K*. *brasiliensis*	*K*. *pinnata*
**PLA**_**2**_		
**1:25 w/w**	31.4 ± 0.1	100 ± 0.0
**1:50 w/w**	83.6 ± 1.5	100 ± 0.0
**1:100 w/w**	100 ± 0.0	100 ± 0.0
**Pre-treatment**		
**Edematogenic**^a^		
**125 mg/kg**	Zero	30.4 ± 10.4*
**250 mg/kg**	11.2 ± 10.5	40.9 ± 7.9**
**500 mg/kg**	23.2 ± 6.3	66.2 ± 8.1***
**MPO**		
**125 mg/kg**	27.0 ± 22.8	77.3 ± 2.2***
**250 mg/kg**	35.3 ± 18.8	58.9 ± 7.3***
**500 mg/kg**	Zero	59.2 ± 4.1***
**Hemorrhagic**		
**125 mg/kg**	30.3 ± 8.5*	48.0 ± 5.3***
**250 mg/kg**	36.7 ± 8.5**	44.3 ± 4.0***
**500 mg/kg**	39.9 ± 4.3**	41.9 ± 4.5***
**Post-treatment**		
**Edematogenic**^a^	19.0 ± 3.5	26.7 ± 5.6*
**MPO**	28.1 ± 11.2	39.8 ± 4.5*
**Hemorrhagic**	1.0 ± 7.7	30.1 ± 5.4*

^a^values from the last time point analyzed (120 min) was considered for calculation.

The values are the mean ± SEM (n = 5).

*p <0.05,

**p<0.01 and

***p<0.001 compared to venom alone (one-way ANOVA followed by the Tukey’s test).

The tests to assess *in vivo* inhibition of local effects induced by *B*. *jararaca venom* were performed in two treatments protocols: pre-treatment, where extract was administered before venom injection and post-treatment, where extract is given to animals after envenomation. The use of medicinal plants as prophylactic agents for snakebites in mice was already demonstrated, for example, by [54]. This study showed that *R*. *alpinia* extracts administered for three days before venom injection inhibited lethal activity of *B*. *asper* venom injected by i.p route in mice. Therefore, the pre-treatment approach could be particularly useful in endemic regions, and by rural workers that are more exposed to snakebites, who could use this resource alternatively and/or complementarily to curative approach. In addition, the use of pre-treatment protocol in pre-clinical assays is useful to avoid false-negative results, that could exists due pharmacokinetics of the drug tested (for example, poor effectiveness because the low absorption rate). By the other hand, the pre-treatment experimental protocol could not reflect, to a certain extent, the extract efficacy when administered after envenoming. Having this in mind, after the evaluation of the efficacy of the extracts in pre-treatment protocol, new experiments were performed to evaluate the efficacy of the extracts when used post-envenomation, in view of to simulate the practical/clinical usage of the plant extract. The post-treatment is an interesting method for evaluating the neutralizing ability of an inhibitor, as it close relates to a practical clinical situation [55, 56]. In this approach, the animals received the extracts 5 min by i.p. route after administration of snake venom.

**Fig 3:**
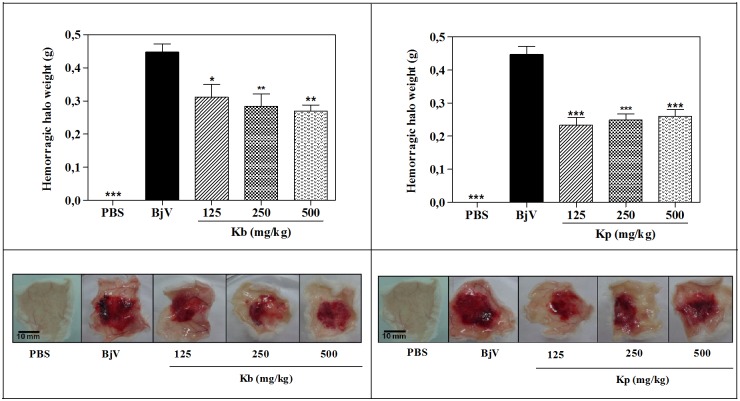
Inhibition of the hemorrhagic activity of *B*. *jararaca* (BjV) venom by extracts of *K*. *brasiliensis* (Kb) (A) and *K*. *pinnata* (Kp) (B) in pre-treatment protocol. BjV was injected s.c. in the ventral region of mice pre-treated i.p. with different doses of extracts. Three hours after venom injection, the skin was removed and weighed. The columns represent the mean ± SEM (n = 5). *<0.05 and **p<0.01 compared to venom alone (one-way ANOVA followed by the Tukey’s test).

The local skin hemorrhage halo is an important effect of bothropic envenomation [26]. Thereby, plants that can reduce the local hemorrhagic action of the venom are of great interest to treat the local effects produced by snakebite. As shown in Fig 3, both extracts, by i.p. route, in pre-treatment, were able to attenuate the hemorrhagic halo produced by venom. As well as for phospholipase activity, *K*. *pinnata* was slightly more active than *K*. *brasiliensis*, mainly regarding the dose 125 mg/kg as the showed in Table 1. In fact, the appearance of the hemorrhagic halo was clearly attenuated, indicating the potentiality of *K*. *brasiliensis* and *K*. *pinnata* leaf extracts in the treatment of local hemorrhage produced by *B*. *jararaca* venom.

**Fig 4:**
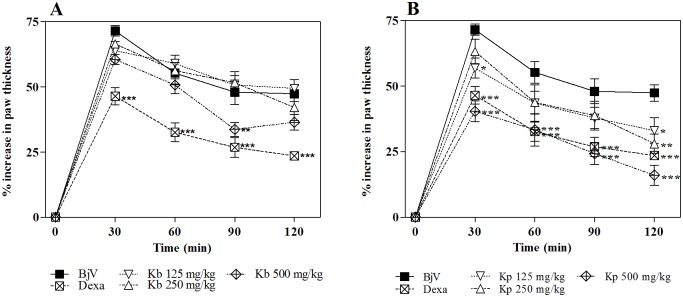
Inhibition of the hemorrhagic activity of *B*. *jararaca* (BjV) venom by extracts of *K*. *brasiliensis* (Kb) (A) and *K*. *pinnata* (Kp) (B) in post-treatment protocol. BjV was injected s.c. in the ventral region of 5 mice before treatement with extract (500 mg/kg, i.p.). Three hours after venom injection, the skin was removed and weighed. The columns represent the mean ± SEM (n = 5). ***p<0.001 compared to venom alone (one-way ANOVA followed by the Tukey’s test).

**Fig 5:**
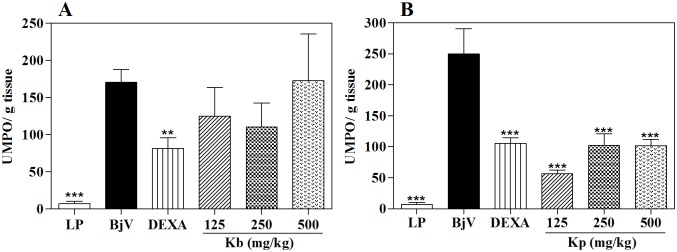
Inhibition of *B*. *jararaca* (BjV) venom-induced hemoglobin accumulation by extracts of *K*. *brasiliensis* (Kb) (A) and *K*. *pinnata* (Kp) (B) in post-treatment protocol. The percentage of activity presented was calculated as: [(Hemoglobin content in animals receiving venom plus extract ÷ Hemoglobin content in animals receiving venom alone) x 100]. Values expressed as mean ± SEM (n = 5). *p<0.05 and ***p<0.001 compared to venom alone (BjV) (100% of activity) (one-way ANOVA followed by the Tukey’s test).

In the post-treatment protocol, no statistic differences between the hemorrhagic halo weight in post-envenomation was observed (Fig 4). By the other hand, the hemoglobin content was significantly decreased around 30% in treated groups with HE of *K*. *pinnata*, as showed in Fig 5. In this protocol, the effect was a little worse probably by pharmacokinetic issues, such as short time for absorption, because the model used has a very fast development. This result reinforces the potentiality of plant species as a promising agent snakebite.

Knowing that local hemorrhage is an important effect of bothropic envenomation, and considering that local hemorrhagic action is produced by the SVMP hemorrhagic (hemorrhagins) action [28], the results suggest that extracts have inhibitory activity upon these toxins. Interesting, the *K*. *pinnata* extract in the post-treatment protocol was able to reduce the hemoglobin content indicating its antihemorrhagic activity. However, it was not able to inhibit the hemorrhagic halo weight. This finding can conduced us to two possibilities: first, perhaps the extract be able to affect the SVMPs non-catalytic sites responsible for adhesives properties affecting mainly the lytic activity of this enzymes per se, and regard the second possibility, is possible that the both methodologies to quantify the hemorrhage (hemorrhagic halo weight and hemoglobin content) showed different sensibility which would entail many different responses.

The anti-hemorrhagic activity of the aqueous extract of *K*. *brasileinsis* leaves pre-incubated with the *B*. *jararaca* venom was described recently, with inhibition of about 40% the diameter of the intradermal hemorrhagic halo [5].

Bothropic envenomation is also characterized by the rapid development of edema and inflammation at the site of venom inoculation [26]. The edematogenic activity is, apparently, a result of a combined action of diverse toxins (Asp49 or Lys49 PLA_2_ and hemorrhagic or non-hemorrhagic snake venom metalloproteinases), rapidly inducing the release of endogenous inflammatory mediators [57]. The modulation by endogenous mediators often reduces the effectiveness of antivenom, since although antivenom can neutralize toxins, it cannot reduce inflammation caused biochemical mediators released by them [33]. Thus, compounds with anti-inflammatory activity are considered potential adjuvants in antivenom therapy for treatment of local effects induced by the bite [58].

**Fig 6:**
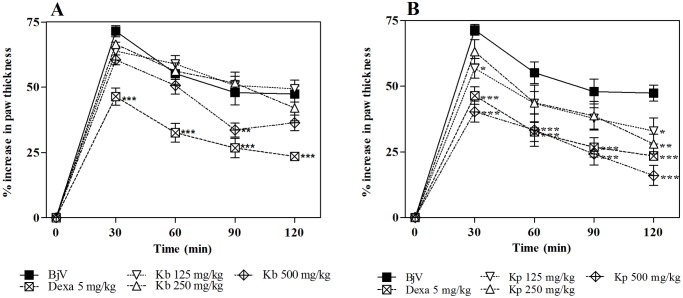
Inhibition of the edematogenic activity of *B*. *jararaca* venom (BjV) by extracts of *K*. *Brasiliensis* (Kb) (A) and *K*. *pinnata* (Kp) (B) in pre-treatment protocol. BjV was injected i.pl. in the right hind paw of mice pre-treated i.p. with different extracts. Paw thickness was measured during 120 min after venom injection. Edema was expressed as the increase in paw thickness calculated as the percentage difference between the paw thickness after (at respective time) and before (basal values) venom injection. The points represent the mean ± SEM (n = 5). *p<0.05, **p<0.01 and ***p<0.001 compared to the group receiving venom alone along with the i.p. injection of 5% castor oil in PBS (Two-way ANOVA followed by the Bonferroni test).

**Fig 7:**
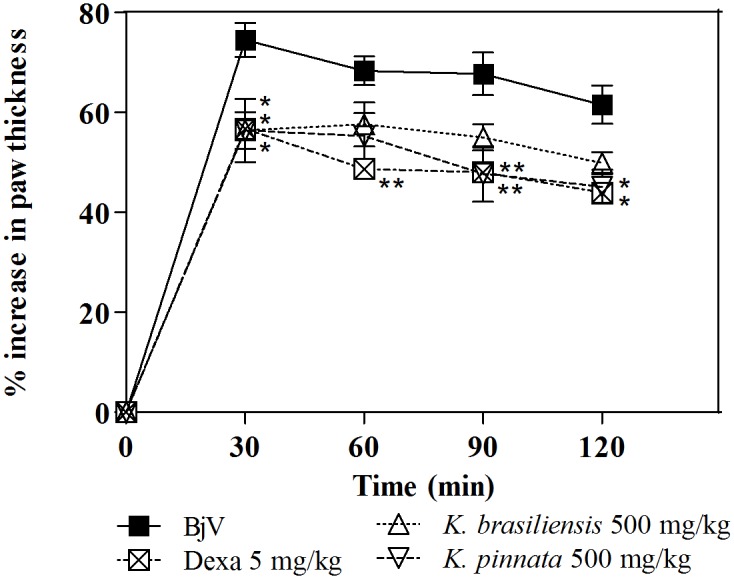
Inhibition of the edematogenic activity of *B*. *jararaca* venom (BjV) by extracts of *K*. *brasiliensis* (Kb) (A) and *K*. *pinnata* (Kp) (B) in post-treatment protocol. BjV was injected i.pl. in the right hind paw of mice 5 min before treatment with extract (500 mg/kg, i.p.). Paw thickness was measured during 120 min after venom injection. Edema was expressed as the increase in paw thickness calculated, by the percentage difference between the paw thickness after (at respective time) and before (basal values) venom injection. The points represent the mean ± SEM (n = 5). *p<0.05 and **p<0.01 compared to the group receiving venom alone along with the i.p. injection of 5% castor oil in PBS (Two-way ANOVA followed by the Bonferroni test).

As can be observed in Fig 6, *K*. *brasiliensis* was not able to inhibit the edematogenic effect induced by *B*. *jararaca*, even at the highest dose tested. On the other hand *K*. *pinnata* (Fig 6) at the highest dose shows significant antiedematogenic effect (p<0.001), with similar effect with the anti-inflammatory drug control dexamethasone (p>0.05). This trend continues with the post-treatment protocol in which *K*. *pinnata* was as effective in inhibiting edema induced by the venom as the standard anti-inflammatory dexamethasone (Fig 7).

**Fig 8:**
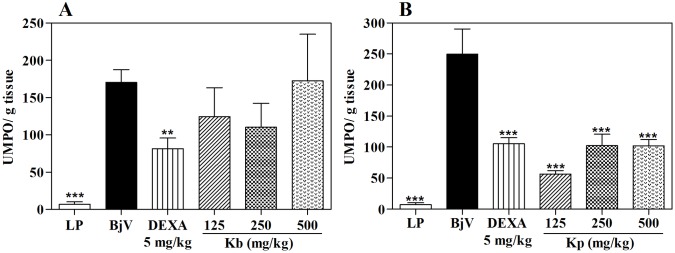
Effect of extracts of *K*. *brasiliensis* (Kb) (A) and *K*. *pinnata* (Kp) (B) on MPO levels in paw edema induced by *B*. *jararaca* venom (BjV) in pre-treatment protocol. LP: left paw (basal control; no injection of venom, extract or dexamethasone). UMPO: a unit of MPO, defined as the equivalent to the consumption of 1μmol of hydrogen peroxide per minute. The columns represent the mean ± SEM (n = 5), **p <0.01 and ***p<0.001 compared to the control group (i.pl. injection of BjV with i.p. administration of 5% castor oil in PBS) after Tukey’s test (one-way ANOVA followed by the Tukey’s test).

**Fig 9:**
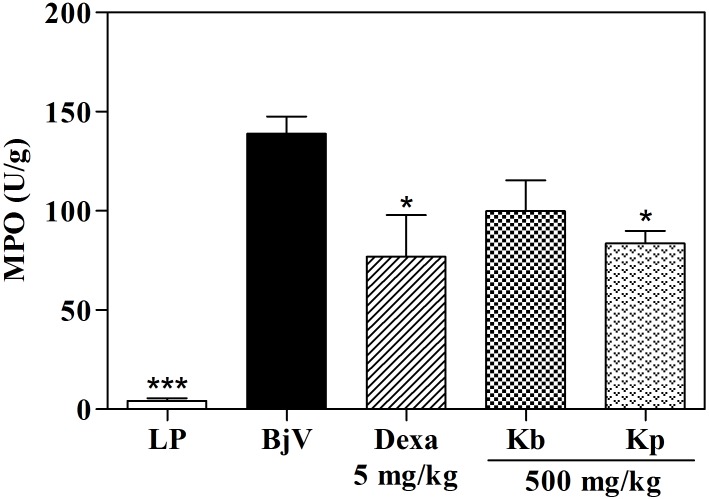
Effect of extracts of *K*. *brasiliensis* (Kb) (A) and *K*. *pinnata* (Kp) (B) on MPO levels in paw edema induced by *B*. *jararaca* venom (BjV) in post-treatment protocol. LP: left paw (basal control; no injection of venom, extract or dexamethasone). UMPO: a unit of MPO, defined as the equivalent to the consumption of 1 μmol of hydrogen peroxide per minute. The columns represent the mean ± SEM (n = 5), *p<0.05 compared to the control group (i.pl. injection of BjV with i.p. administration of 5% castor oil in PBS) (one-way ANOVA followed by the Tukey’s test).

The MPO analysis, in the highest tested dose, *K*. *pinnata* was able to inhibit 55.4% of the MPO activity, while HE of *K*. *brasiliensis*, in all doses assayed, did not produce significant effects (Fig 8 and Table 1). To the same occurred in the post-treatment protocol, in which *K*. *pinnata* was as effective in inhibiting the MPO by the venom as the standard treatment (Fig 9), corroborating the percentage dencrease in paw thickness. This significant inhibition of MPO activity indicates that the antiedematogenic effect presented by the extract could be related with an inhibition of cell migration, since this enzyme is a quantitative marker of inflammatory cell influx to paw tissue injected with venom [43, 59].

Regarding *K*. *pinnata*, the anti-inflammatory activity here observed corroborate with the literature, in which the aqueous and ethanol extracts of *K*. *pinnata* were found to be effective in inhibiting the paw edema induced by carrageenan and the ear edema induced by croton oil [14, 15]. Besides flavonoids, other plant secondary metabolites have been identified and isolated from *K*. *pinnata*, e.g., terpenes, steroids and bufadienolides. Thus, the *K*. *pinnata* anti-inflammatory activity has been attributed to synergism among flavonoids, triterpenes, and steroids [15].

According to the literature, the treatment of animals with the juice obtained from the leaves of *K*. *brasiliensis* reduced the paw thickness, leukocyte infiltration, and blood flow in the paw area in the zymosan-induced inflammation [10]. Another study showed that the aqueous extract of *K*. *brasiliensis* was effective in inhibiting the paw edema induced by carrageenan [19]. A study conducted by Fonseca et al. [11] showed that the topical application of an aqueous extract of the aerial parts of *K*. *brasiliensis* in mice significantly attenuated the edematogenic, necrotic and hemorrhagic activities of *Bothrops alternatus* venom. However, our results with *K*. *brasiliensis* showed that it does not have significant antiedematogenic activity against *B*. *jararaca* snake venom, even in the highest dose. Some factors may be interfering with the action of the extract against the edema, such as extract type (previous studies employed the juice or aqueous extracts of the leaves, while in the present work a hydroethanolic extract was used) and route of administration used (some studies employed an oral or topical route, while in the present work the intraperitoneal route was used).

In our study, the chemistry profile reveals that flavonoids are the major secondary metabolites in raw material and extracts from both *K*. *brasiliensis* and *K*. *pinnata*. According to the literature, the presence of flavonoids could be especially interesting in antiophidic plants since these compounds are able to promote strong hydrogen bonds with amides of protein chains and exhibit metal chelating activity, as well as present potent anti-inflammatory and antioxidant activities [28, 36]. In fact, several studies show that flavonoids (such as apigenin, moreloflavone, quercetin, luteolin, among others) have the ability to inhibit SVMPs, PLA_2_s, and hyaluronidases purified from snake venoms, showing great inhibitory effects against envenoming-induced local effects [60, 61, 62, 63, 64, 65]. So, the great amounts of flavonoids in raw materials and extracts of *K*. *brasiliensis* and *K*. *pinnata* extracts could justify, at least partially, the inhibitory activities presented by these extracts. However, considering that extracts are a complex mixture of miscellaneous chemical groups, many other active constituents in these extracts may be involved, acting by different mechanisms against several different toxins in snake venoms. In fact, in many cases, the whole herbal extracts are more powerful than the herbal compounds [56]. So, further studies are needed verify this in the studied species.

## Conclusion

In summary, the results presented in this work demonstrated the potentiality of *K*. *pinnata* extract as an antiophidic agent, particularly against local effects induced by *B*. *jararaca* envenomation. *K*. *brasiliensis*, a very close vegetal species that is even used in traditional medicine interchangeably with *K*. *pinnata* as antiophidic, by the other hand, presented only discreet or even absent inhibitory efficiency against the same effects induced by *B*. *jararaca* venom. So, this study justifies the inclusion of *K*. *pinnata* in RENISUS and reinforce its potentiality for the generation of pharmaceutical products, as it shows satisfactory results against local effects induced by *B*. *jararaca* venom.

Further studies aiming at the complete isolation of the biological active compounds of the extract, especially flavonoids are ongoing.

In conclusion, our results demonstrated the potential antiophidic activity of *K*. *pinnata*, suggesting its potential as a source of bioactive molecules as well as its potential usefulness as an adjuvant therapy against *Bothrops*-induced local inflammatory and hemorrhagic lesions.

## Supporting Information

S1 Fig Leaves and inflorescences of *Kalanchoe brasiliensis* Cambess (Crassulaceae) plant (A) and *Kalanchoe pinnata* Lamarck Persoon (Crassulaceae) plant (B). Photography by Júlia Morais Fernandes.
(TIF)

S2 Fig TLC of *Kalanchoe brasiliensis* (A) and *Kalanchoe pinnata* (B) HE extracts. Mobile phase: (A-1 and B1) toluene: ethyl acetate: formic acid (5:5:0.5, v/v/v); (A-2 and B-2): ethyl acetate: formic acid: methanol: water (10:0.5:0.6:0.2, v/v/v/v). Stationary phase: silica gel 60 F254. Detection with natural product A reagent and visualization under UV light (365 nm). CH_2_Cl_2_ = dichloromethane fraction, EtAcO = ethyl acetate fraction, BuOH = *n*-butanol fraction.
(TIF)

S1 Table Compounds identified by HPLC-DAD-MS in HE extract of the leaves of *Kalanchoe brasiliensis* species. RT, retention time. *The intensity of each peak as a percentage, is provided adjacent to the corresponding m/z ration. ^#^Major peak.
(PDF)

S2 Table Compounds identified by HPLC-DAD-MS in HE extract of the leaves of *Kalanchoe pinnata* species. RT, retention time. *, The intensity of each peak as a percentage, is provided adjacent to m/z ration of the same. ^#^, Major peak. CAF, cafeoil. sh, Shoulder in the UV spectrum.
(PDF)

S1 File **Supplementary material:** Scheme 1. MS3 ion trap analysis to identify the aglycone moiety at m/z 333. Scheme 2. MS3 ion trap analysis to identify the aglycone moiety at m/z 317.
(DOCX)
