# Correction: Inhibitory Effects of Hydroethanolic Leaf Extracts of *Kalanchoe brasiliensis* and *Kalanchoe pinnata* (Crassulaceae) against Local Effects Induced by *Bothrops jararaca* Snake Venom

**DOI:** 10.1371/journal.pone.0172598

**Published:** 2017-02-16

**Authors:** Júlia Morais Fernandes, Juliana Félix-Silva, Lorena Medeiros da Cunha, Jacyra Antunes dos Santos Gomes, Emerson Michell da Silva Siqueira, Luisa Possamai Gimenes, Norberto Peporine Lopes, Luiz Alberto Lira Soares, Matheus de Freitas Fernandes-Pedrosa, Silvana Maria Zucolotto

The image for Fig 4 is a duplicate of the image for Fig 6. Please see the correct Fig 4 here.

**Fig 4:**
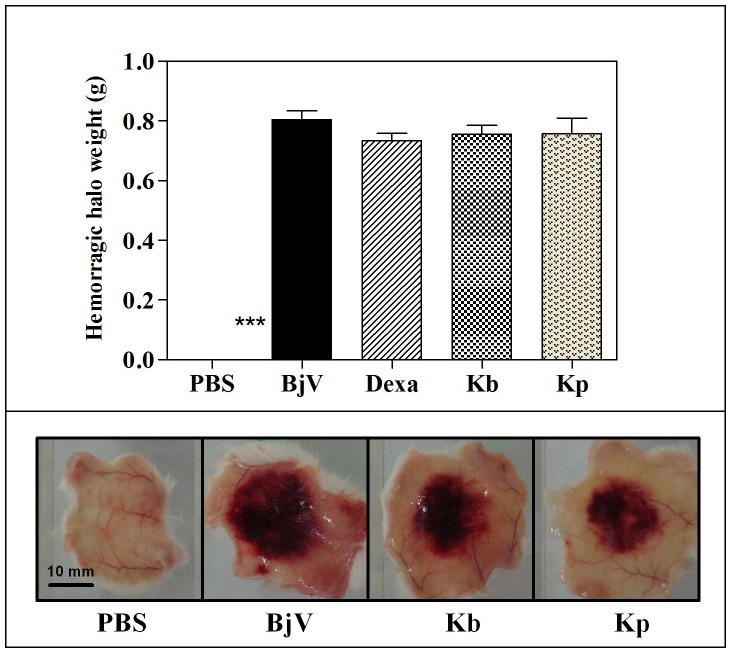
Inhibition of the hemorrhagic activity of *B*. *jararaca* (BiV) venom by extracts of *K*. *brasiliensis* (Kb) (A) and *K pinnata* (Kp) (B) in post-treatment protocol. BjV was injected s.c. in the ventral region of 5 mice before treatement with extract (500 mg/kg, i.p.). Three hours after venom injection, the skin was removed and weighed. The columns represent the mean ± SEM (n = 5). ***p<0.001 compared to venom alone (one-way ANOVA followed by the Tukey’s test).

The image for Fig 5 is a duplicate of the image for Fig 8. Please see the correct Fig 5 here.

**Fig 5:**
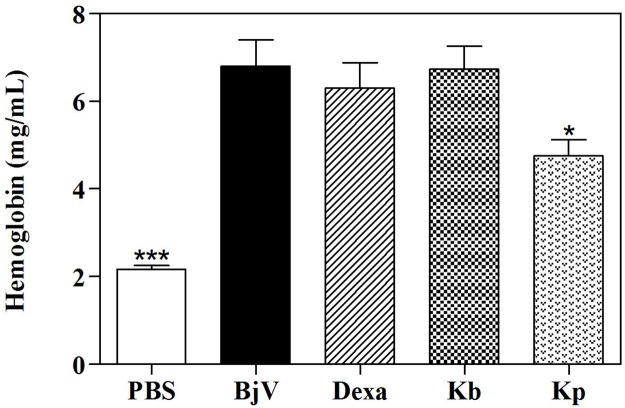
Inhibition of *B*. *jararaca* (BjV) venom-induced hemoglobin accumulation by extracts of *K*. *brasiliensis* (Kb) (A) and *K*. *pinnata* (Kp) (B) in post-treatment protocol. The percentage of activity presented was calculated as: [(Hemoglobin content in animals receiving venom plus extract ÷ Hemoglobin content in animals receiving venom alone) x 100]. Values expressed as mean ± SEM (n = 5). *p<0.05 and ***p<0.001 compared to venom alone (BjV) (100% of activity) (one-way ANOVA followed by the Tukey’s test).

In the figure title of Fig 6, the word *Brasiliensis* should be lowercase. Please see the corrected caption here.

**Fig 6:**
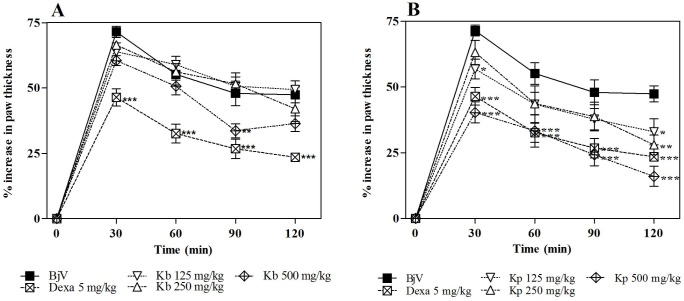
Inhibition of the edematogenic activity of *B*. *jararaca* venom (BjV) by extracts of *K*. *brasiliensis* (Kb) (A) and *K*. *pinnata* (Kp) (B) in pre-treatment protocol. BjV was injected i.pl. in the right hind paw of mice pre-treated i.p. with different extracts. Paw thickness was measured during 120 min after venom injection. Edema was expressed as the increase in paw thickness calculated as the percentage difference between the paw thickness after (at respective time) and before (basal values) venom injection. The points represent the mean ± SEM (n = 5). *p<0.05, **p<0.01 and ***p<0.001 compared to the group receiving venom alone along with the i.p. injection of 5% castor oil in PBS (Two-way ANOVA followed by the Bonferroni test).

The image for the S1 Fig is incorrect. Please see the correct image for the S1 Fig here.

## Supporting information

S1 Fig Leaves and inflorescences of *Kalanchoe brasiliensis* Cambess (Crassulaceae) plant (A) and *Kalanchoe pinnata* Lamarck Persoon (Crassulaceae) plant (B). Photography by Júlia Morais Fernandes. doi:10.1371/journal.pone.0168658.s001.
(JPG)
