# Supplementary figures and images for: Inhibitory Effects of Hydroethanolic Leaf Extracts of Kalanchoe brasiliensis and Kalanchoe pinnata (Crassulaceae) against Local Effects Induced by Bothrops jararaca Snake Venom
Source: PLoS One. 2016 Dec 29;11(12):e0168658. doi: 10.1371/journal.pone.0168658 (PMC5199091; doi:10.1371/journal.pone.0168658)

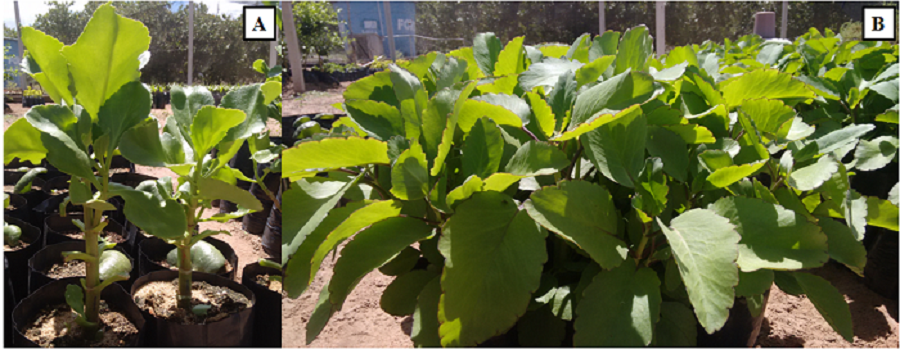

Supplement: S1 Fig — Photography by Júlia Morais Fernandes. (TIF) [file pone.0168658.s001.tif]

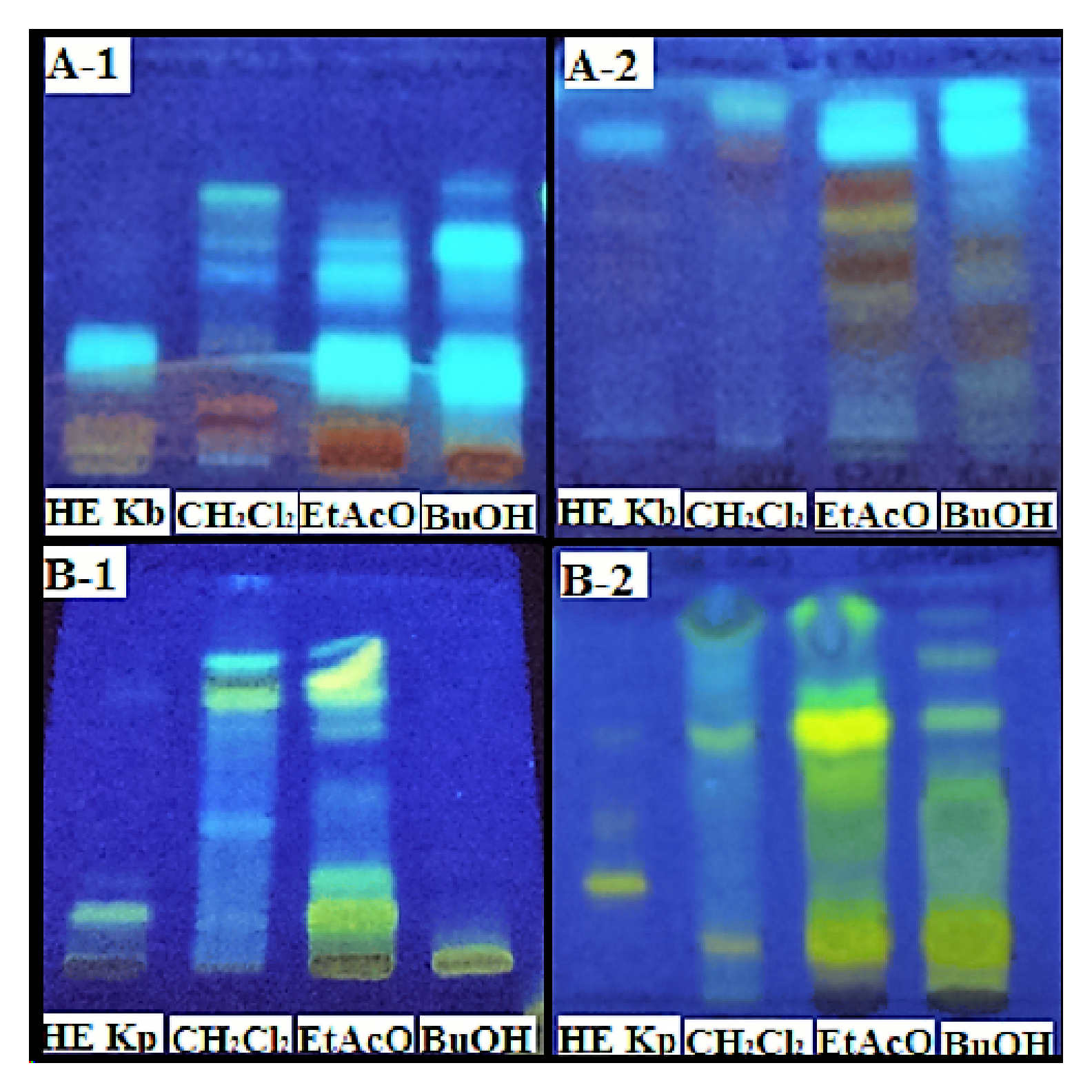

Supplement: S2 Fig — Mobile phase: (A-1 and B1) toluene: ethyl acetate: formic acid (5:5:0.5, v/v/v); (A-2 and B-2): ethyl acetate: formic acid: methanol: water (10:0.5:0.6:0.2, v/v/v/v). Stationary phase: silica gel 60 F254. Detection with natural product A reagent and visualization under UV light (365 nm). CH2Cl2 = dichloromethane fraction, EtAcO = ethyl acetate fraction, BuOH = n-butanol fraction. (TIF) [file pone.0168658.s002.tif]
