# Supplementary material for: Inhibitory Effects of Hydroethanolic Leaf Extracts of Kalanchoe brasiliensis and Kalanchoe pinnata (Crassulaceae) against Local Effects Induced by Bothrops jararaca Snake Venom
Source: PLoS One. 2016 Dec 29;11(12):e0168658. doi: 10.1371/journal.pone.0168658 (PMC5199091; doi:10.1371/journal.pone.0168658)
Supplement: S1 Table — RT, retention time. *The intensity of each peak as a percentage, is provided adjacent to the corresponding m/z ration. #Major peak. (PDF) [file pone.0168658.s003.pdf]

S1 Table. Compounds identified by HPLC-DAD-MS in HE extract of the leaves of *Kalanchoe brasiliensis* species.

Peak	TR (min)	MS*(m/z)**	MS²*(m/z)**	MS³*(m/z)**	UV max (nm)	Compound
**1**	15.3	$[M+H]^+$ 787(3) $[MH-(deoxy\text{-}hexose)]^+$ 641(17) [MH-(2deoxy-hexoses)] 495(2) $[aglicon]^+$ 333(4)	787→641(32); 495(78); 333(100).	333→ 318(100); 301(100); 290(38); 272(32); 245(15); 216(6,3); 186(12); 169(79); 142(23); 111(4,8).	268, 340.	Patuletin-*O*-hexoside-di-*O*-deoxy-hexoside
**2**	18.3	$[M+H]^+$ 787(15) $[MH-(deoxy\text{-}hexose)]^+$641(27) $[MH-(deoxy\text{-}hexose]^+$495(9) $[aglicon]^+$333(1)	787→641(30); 605(1,2); 495(33); 479(9); 333(100); 318(5).	333→ 318(100); 301(100); 290(38); 272(32); 245(15); 216(6,3); 186(12); 169(79); 142(23); 111(4,8).	268, 350.	Patuletin-*O*-hexoside-di-*O*-deoxy-hexoside
**3**	19.1	$[M+H]^+$ 771(6.8) $[MH-(deoxy\text{-}hexose)]^+$ 625(44.6) [MH-(2deoxy-hexoses)] 479(4,22) $[aglicon]^+$ 317(3.5)	771→625(49.6); 479(100); 317(55.6); 302(9.8).	317→302(100); 285(100); 274(43.6); 273(50.4); 257(24.9); 256(48); 228(71.7); 214(21.9); 203(20); 168(41.2); 142(28); 134(31); 107(18.5).	268, 314.	Eupafolin-*O*-hexoside-di-*O*-deoxy-hexoside
**4**	19.7	$[M+H]^+$ 641(35,7) $[MH-(hexose)]^+$ 479(3) $[aglicon]^+$ 333(0.2)	641→ 495(6,6); 479(78); 333(100); 318(3.7).	333→318(100); 301(100); 290(38); 272(32); 245(15); 216(6.3); 186(12); 169(79); 142(23); 111(4.8).	268, 359.	Patuletin-*O*-hexoside-*O*-deoxy-hexoside
**5**	20.3	$[M+H]^+$ 787(100) $[MH-(deoxy\text{-}hexose)]^+$ 641(23.5) [MH-(2deoxy-hexoses)] 495(11,7) $[aglicon]^+$ 333(2.1)	787→641(83.8); 495(100); 333(87.4).	333→ 318(100); 301(100); 169(51.85).	268, 350.	Patuletin-*O*-hexoside-di-*O*-deoxy-hexoside
**6**[#]	23.3	$[M+H]^+$ 625(92) $[MH-(hexose)]^+$ 463(23.5)	625→463(84); 317(100).	317→ 302(100); 285(100); 274(26.5); 266(13.8); 257(13); 255(24.6); 246(26); 232(30.4); 228(100); 218(12); 107(29).	268, 340.	Eupafolin-*O*-hexoside-*O*-deoxy-hexoside
**7**[#]	24.7	$[M+H]^+$ 625(73) $[MH-(deoxy\text{-}hexose)]^+$ 479(32) [MH-(2deoxy-hexose)] 333(9.5)	625→ 479(57.5); 333(31.6); 317(100)	333→318(100); 301(100); 290(38); 272(32); 245(15);	259, 354.	Patuletin-di-*O*-deoxy-hexoside

				216(6,3); 186(12); 169(79); 142(23); 111(4.8).		
**8**#	25.4	[M+H]+787(2) [MH-(deoxy-hexose)]+641(100) [MH-(2deoxy-hexose)]+495(19,2) [aglicon] + 333(3)	787→ 641(66.8); 495(100); 479(19.9); 333(74.2).	333→318(100); 301(90); 290(41.5); 272(71.4); 263(13.5); 244(38.0); 227(15); 169(100); 168(91.8); 142(33.5).	268, 350.	Patuletin-*O*-hexoside-di-*O*-deoxy-hexoside
**9**	26.1	[M+H]+ 611(100) [MH- (deoxy-hexose)]+ 465(18.6) [aglicon] + 303(3)	611→465(58.7); 449(32); 303(100).	303→ 285(49.5); 261(1.3); 257(100); 247(26.6); 229(43); 165(45); 153(8); 137(4.5), 121(6.2); 111(4.8).	268, 340.	Quercetin-*O*-hexoside-*O*-deoxy-hexoside
**10**	27.4	[M+H]+ 829(100) [MH-(deoxy-hexose)]+ 683(23.5) [MH-(acetyl-deoxy-hexose)]+ 641(12) [MH-346]+ 483(69.1) [MH-362]+ 467(6.7)	829→811(11.1); 683(30.5); 667(4.6) 641(43.4); 495100); 479(12.6); 333(85).	333→ 318(100); 301(100); 290(38); 272(32); 245(15); 216(6.3); 186(12); 169(79); 142(23); 111(4,8).	268, 350.	Patuletin-*O*-hexoside-*O*-acetyl- deoxy-hexoside-*O*-deoxy-hexoside
**11**#	29.6	[M+H]+609(100) [MH-(deoxy-hexose)]+ 463(26) [aglicon]+317(7.3) [MH-306]+303(17.9)	609→ 463(100); 317(69.5).	317→ 302(100); 285(100); 274(35); 273(39,7); 266(13.8); 257(13); 255(24.6); 246(26); 232(30.4); 228(100); 218(12); 107(29).	268, 350.	Eupafolin-di-*O*-deoixy-hexosíde
**12**#	31.7	[M+H]+829(38,2) [MH-(deoxy-hexose)]+ 683(11.6)	829→683(49); 641(35.7); 495(100); 333(82.3).	333→ 318(100); 301(100); 290(38); 272(32); 245(15); 216(6.3); 186(12); 169(79); 142(23); 111(4.8).	312.	Patuletin-*O*-hexoside-*O*-acetyl- deoxy-hexoside-*O*-deoxy-hexoside
**13**#	-	-	-	-	-	Compound not didentifie
**14**#	35.1	[2M+Na]+ 979(4,2) [M+H]+479(77) [MH-(deoxy-hexose)]+ 333(100)	479→333(100)	333→318(100); 301(98); 290(29.4); 272(15.5); 244(13); 186(8); 169(100); 142(8).	268. 330.	Patuletin-*O*-deoxy-hexoside

**15**#	36.5	[M+H]+ 667(100)<br>[MH-(deoxy-hexose)]+ 521(40)<br>[aglicon] + 333(6)	667→ 521(100); 479(3.6); 333(33.5).	333→ 318(100); 301(95.7); 289(100);244(47.3); 202(21.2); 186(59.2); 169(19); 142(27.7); 123(29.4).	268, 350.	Patuletin-*O*-deoxy-hexoside-*O*-acetyl-deoxy-hexoside
**16**#	38.3	[M+H]+ 667(100)<br>[MH-(acetyldeoxy-hexose)]+ 479(40)<br>[aglicon] + 333(4,7)	667→ 521(6.7); 479(61.3); 333(100).	333→ 318(100); 301(100); 290(38.7); 289(43.7); 272(49.4); 244(25.4); 234(12.8);186(13.4); 169(83.1); 142(22.8).	268, 350.	Patuletin-*O*-deoxy-hexoside-*O*-acetyl-deoxy-hexoside
**17**#	39.3	[M+H]+ 667(100)<br>[MH-(deoxy-hexose)]+ 521(46)<br>[aglicon] + 333(4.4)	667→ 521(100); 479(3.5); 333(59).	333→ 318(100); 301(100); 290(19.2); 289(20.9); 273(22.4); 272(16.2); 245(16); 244(16.3); 186(16); 169(52.7); 151(13.6); 142(13.6).	268, 350.	Patuletin-*O*-deoxy-hexoside-*O*-acetyl-rhamnoside
**18**	42.7	[M+H]+ 651(100)<br>[MH-(desóxi-hexose)]+ 505(29.5)<br>[aglicon] + 317(7.2)	651→ 505(100); 463(5); 317(42)	317→ 302(100); 285(100); 274(43.6); 273(50.4); 257(24.9); 256(48); 228(71.7); 214(21.9); 203(20); 168(41.2); 142(28); 134(31); 107(18.5).	268, 350.	Eupafolin-*O*-deoxy-hexoside-*O*-acetyl-deoxy-hexoside
**19**	46.3	[M+H]+ 651(100)<br>[MH-(acetyl-deoxy-hexose)]+ 463(4.8)<br>[aglicon] + 317(4.4)	651→ 505(100); 463(3.7); 317(29.2).	317→ 302(100); 285(100); 274(43.6); 273(50.4); 257(24.9); 256(48); 228(71.7); 214(21.9); 203(20); 168(41.2); 142(28); 134(31); 107(18.5).	269, 350.	Eupafolin-*O*-rhamnoside-*O*-acetyl-rhamnoside
**20**	47.1	[M+H]+ 667(100)<br>[MH-(deoxy-hexose)]+ 521(29)<br>[aglicon] + 333(78)	667→ 521(100); 479(2.5); 333(78).	333→ 318(100); 301(100); 290(30.9); 289(11.9); 262(26); 244(31.2); 169(42.3).	269, 350.	Patuletin-*O*-deoxy-hexoside-*O*-acetyl-deoxy-hexoside
**21**	50.6	[M+H]+ 709(100)<br>[MH- (acetyl-deoxy-hexose)]+ 521(4.2)<br>[MH-226]+ 483(2.7)	709→521(100); 333(48).	333→318(100); 301(100); 290(30.9); 289(11.9);	269, 355.	Patuletin-di-*O*-acetyl-deoxy-hexosíde

		$[MH-242]^+$ 467(1.4)		262(26); 244(31.2); 169(42.3).		
**22#**	53.9	$[M+H]^+$ 709(100) [MH- (acetyl-deoxy-hexose)]$^+$ 521(9)	709→521(100); 333(79.4).	333→ 318(100); 301(100); 290(38); 272(32); 245(15); 216(6.3); 186(12); 169(79); 142(23); 111(4.8).	269, 355.	Patuletin-di-*O*-acetyl-deoxy-hexoside
**23#**	58.0	$[M+H]^+$ 709(100) [MH- (acetyl-deoxy-hexose)]$^+$ 521(10)	709→521(100); 333(85.8).	333→ 318(100); 301(83.9); 290(78.6); 289(18.5); 272(19.5); 1699 (100); 151(32).	269, 355.	Patuletin-di-*O*-acetyl-deoxy-hexoside
**24**	63.1	$[M+H]^+$ 709(100) [MH- (acetyl-deoxy-hexose)]$^+$ 521(5,4) [aglicon]$^+$ 333(4.8)	709→521(100); 506(7.6); 412(6);333(83.3).	333→ 318(100); 301(83.9); 290(78.6); 289(18.5); 272(19.5); 169(100); 151(32).	269, 355.	Patuletin-di-*O*-acetyl-deoxy-hexoside
**25**	63.3	$[M+H]^+$693(100) [MH-(acetyl- deoxy-hexose)]$^+$ 505(6)	693→ 505(100); 317(66.7).	317→ 302(100).	269, 355.	Eupafolin-di-*O*-acetyl- deoxy-hexoside
**26**	65.3	$[M+H]^+$693(100) [MH-(acetyl-deoxy-hexose)]$^+$ 505(6)	693→ 505(100); 317(74.9).	317→ 302(100).	267, 317, 355.	Eupafolin-di-*O*-acetyl-deoxy-hexoside
**27**	65.7	$[M+H]^+$693(100) [MH-(acetyl-deoxy-hexose)]$^+$ 505(7)	693→ 547(14,8); 505(75.8); 317(100).	317→ 302(100).	267, 317, 355.	Eupafolin-di-*O*-acetyl-deoxy-hexoside
**28**	66.2	$[M+H]^+$751(100) [MH-(acetyl-deoxy-hexose)]$^+$ 563(12,5) [MH-di-acetyl-deoxy-hexose]$^+$ 521(7,5) [aglicon]$^+$ 333(70,5)	751→ 563(100); 521(7.5); 333(70.5).	333→ 318(100); 301(100); 290(38); 272(32); 245(15); 216(.,3); 186(12); 169(79); 142(23); 111(4.8).	268, 350.	Patuletin-*O*-acetyl-deoxy-hexoside-*O*-di-acetyl-deoxy-hexoside.

RT, retention time. *, The intensity of each peak as a percentage, is provided adjacent to m/z ration of the same. #, Major peak.
