# Supplementary material for: Inhibitory Effects of Hydroethanolic Leaf Extracts of Kalanchoe brasiliensis and Kalanchoe pinnata (Crassulaceae) against Local Effects Induced by Bothrops jararaca Snake Venom
Source: PLoS One. 2016 Dec 29;11(12):e0168658. doi: 10.1371/journal.pone.0168658 (PMC5199091; doi:10.1371/journal.pone.0168658)
Supplement: S2 Table — RT, retention time. *, The intensity of each peak as a percentage, is provided adjacent to m/z ration of the same. #, Major peak. CAF, cafeoil. sh, Shoulder in the UV spectrum. (PDF) [file pone.0168658.s004.pdf]

S2 Table. Compounds identified by HPLC-DAD-MS in HE extract of the leaves of *Kalanchoe pinnata* species.

Peak	TR (min)	MS (*m/z*)	MS² (*m/z*)	MS³ (*m/z*)	UV max (nm)	Compound
**1**	7.2	$[M-H]^-$ 353 (18)<br>$[M-CAF]^-$ 191 (100)<br>$[M-CAF-H_2O]^-$ 173 (71.7)	353→ 173 (100); 111 (18.7).	__________	__________	3-*O*-caffeoylquinic Acid
**2**	8.7	$[M-H]^-$ 353 (2.6)<br>$[M-CAF]^-$ 191 (100)<br>$[M-CAF-H_2O]^-$ 173 (46.7)	353→ 173(100); 111(17.5).	__________	__________	5-*O*-caffeoylquinic Acid
**3**	9.1	$[M-H]^-$ 353 (1)<br>$[M-CAF]^-$ 191 (100)<br>$[M-CAF-H_2O]^-$ 173 (18.7)	353→ 173(100); 111(18).	__________	___________	4-*O*-caffeoylquinic Acid
**4**#	16.8	$[M+H]^+$611 (100)<br>$[MH-(deosy-hexose)]^+$465 (35)<br>$[MH-272]^+$339(24)<br>$[MH-464]^+$147(21)	611→ 465(100); 303(28).	303→ 285(62.9); 275(15.4); 257(100); 247(34.7); 229 (47.8); 213(6,2); 195 (7.7); 183 (10); 165 (71.2); 153 (14.4); 137 (17,2); 121 (5.6); 11 (6.4)	312	Quercetin-*O*-deoxy-hexoside-*O*-hexoside
**5**	24.5	$[M+H]^+$ 597(10.8)<br>$[MH-132]^+$ 465(50.7)<br>$[MH-278]^+$ 319(50.7)	597→ 465 (100); 447 (16.7); 429 (18.5); 361 (13.5); 319 (63.5).	319→ 301 (96.6); 283(18.13); 273(100); 263(9.4); 245 (9.3); 207 (4.4); 195 (16.1); 165 (36.3); 153 (12.8); 127 (5.31).	275, 370sh.	

		$[MH-430]^+$ 181(24.8)<br>$[MH-512]^+$ 99(10.6)				
**6**	29.8	$[M+H]^+$641 (100)<br>$[MH-(deoxy-hexose)]^+$495 (43.7)<br>$[MH-272]^+$339(32)<br>$[aglycone]^+$303(10.8)	641→ 495(100); 479(12.2) 333(64).	333→318(100); 301(100); 290 (38); 272 (32); 245 (15); 216 (6.3); 186 (12); 169 (79); 142 (23); 111 (4.8).	269, 320, 360.	Patuletin-*O*-deoxy-hexoside-*O*-hexoside.
**7**	30.7	$[M+H]^+$ 597(100)<br>$[MH-132]^+$ 465 (13.3)<br>$[MH-278]^+$ 319 (83.4)	597→ 465(100); 447(11); 429(10); 361(6); 319(32).	319→301 (96.6); 283 (18.13); 273(100); 263(9.4); 245 (9.3); 207 (4.4); 195(16.1); 165(36.3); 153(12.8); 127 (5.31).	269,350.	
**8**	32.7	$[M+H]^+$ 465(70,9)<br>$[MH-(hexose)]^+$ 303(68)	465→ 303(100).	303→ 285 (66.5); 275 (20.5); 257(100); 247(38.6); 229(87,8); 165(55.4); 153 (22.8); 137 (12.34); 121 (5.2); 111 (5.6).	269, 350.	Quercetin-*O*-hexoside
		$[M+H]^+$ 595 (18,4)<br>$[MH-(deoxy-hexose)]^+$ 449 (100)	449→287(100)	287→ ---------		Kaempferol-*O*-deoxy-hexoside-*O*-hexoside.
**9**	36.4	$[M+H]^+$ 611 (18,4)<br>$[MH-(pentose)]^+$ 479 (5,4)	611→ 479(39.6); 461 (13); 443 (21.3); 375 (8.7); 333(100).	333→ ---------	269, 355.	Patuletin-*O*-deoxy-hexoside –*O*-pentoside

		[MH-278]$^{+}$ 333 (100)				
**10**	37.0	[M+H]$^{+}$ 463(100)<br>[MH-130]$^{+}$ 333(3)	463→ 287 (100)	287→ ---------	268, 355.	Kaempferol
**11**#	37.8	[M+H]$^{+}$581 (100)<br>[M-(pentose)]$^{+}$449 (7,8)<br>[aglicona]$^{+}$ 303 (77)	581→ 449(100); 431(10,5); 413(15,8); 345(6,5); 303(44,3).	303→ 285(42,7); 275(13,3); 274(8,7); 257(100); 247(25,2); 229(48,8); 165(59,4); 153(12,4); 149(9,1); 137(8,7); 121(10); 111(10,3).	255, 348	Quercetin-*O*-deoxy-hexoside-*O*-pentoside
**12**#	39.3	[M+H]$^{+}$581 (75)<br>[M-(pentose)]$^{+}$449 (23,5)<br>[MH-241]$^{+}$340 (18)<br>[aglycone]$^{+}$ 303 (100)	581→ 449 (91.8); 345 (5.2); 303(100).	303→ 285(42.7); 275 (13.3); 274 (8.7); 257(100); 247(25.2); 229(48.8); 165(59.4); 153 (12.4); 149 (9.1); 137(8.7); 121(10); 111.	269, 350	Quercetin-*O*-deoxy-hexoside-*O*-pentoside
**13**#	39.6	[M+H]$^{+}$581 (14,4)<br>[M-(pentose)]$^{+}$449(35.7)<br>[aglycone]$^{+}$ 303(100)	581→ 449(100); 431(9.7); 413 (19.3); 345 (10.5); 303(48)	303→ 285(49.4); 274 (17); 257 (100); 247 (33.6); 229 (69); 165 (45.7); 153 (20.9); 137 (15); 121 (8.3).	269, 350	Quercetin-*O*-deoxy-hexoside-*O*-pentoside
**14**	40.6	[M+H]$^{+}$ 609(100)<br>[aglycone]$^{+}$ 303(6)	609→ 477 (41.6); 301(100).	301→	269, 350.	
**15**	43.3	[M+H]$^{+}$ 493(100)<br>[M-hexose]$^{+}$ 331(4)	493→ 331(100).	331→	269, 350.	

**16**	44.8	[M+H]+ 565(100)<br><br>[aglycone]+ 287 (72.7)	565→ 433 (100); 397 (16.7); 329 (4.4);287(35.3).	287→ 269 (13); 241(78.4); 231(15.7); 213(71); 203 (18.3); 183 (34); 165 (100); 153 (18.2); 133 (21.5); 121 (26.4).	264, 355.	Kaempferol-*O*-deoxy-hexoside-*O*-pentoside
**17**	45.4	[M+H]+ 595(100)<br><br>[MH-122]+ 473 (33.2)<br><br>[aglycone]+ 317(84,7)	595→ 463 (100); 427 (21); 383 (5.8); 317 (67.2)	317→ 302(100); 285 (39.4); 274 (5.7); 271(11.6); 257 (10.5); 261 (9.3); 243(5); 165 (8.7); 153 (5.3); 139 (14.7).	266, 350.	Eupafolin-*O*-deoxy-hexoside-*O*-pentoside
**18**	47.0	[M+H]+ 477(100)	477→ 301(100)	301→ 286(100); 283 (8.5); 273 (17.9); 245(7.4); 205(8.2).	269, 350.	
**19**	49.0	[M+H]+ 507(100)	507→ 331(100)	331→316(100).	269, 355.	

RT, retention time. *, The intensity of each peak as a percentage, is provided adjacent to m/z ration of the same. #, Major peak.
CAF, cafeoil. sh, shoulder in the UV spectrum.
