# Supplementary material for: Inhibitory Effects of Hydroethanolic Leaf Extracts of Kalanchoe brasiliensis and Kalanchoe pinnata (Crassulaceae) against Local Effects Induced by Bothrops jararaca Snake Venom
Source: PLoS One. 2016 Dec 29;11(12):e0168658. doi: 10.1371/journal.pone.0168658 (PMC5199091; doi:10.1371/journal.pone.0168658)
Supplement: S1 File — Supplementary material: Scheme 1. MS3 ion trap analysis to identify the aglycone moiety at m/z 333. Scheme 2. MS3 ion trap analysis to identify the aglycone moiety at m/z 317. (DOCX) [file pone.0168658.s005.docx]

**Scheme 1. MS3 ion trap analysis to identify the aglycone moiety at m/z 333.**

**Scheme 2. MS3 ion trap analysis to identify the aglycone moiety at m/z 317.**
