# Supplementary figures and images for: Correction: Inhibitory Effects of Hydroethanolic Leaf Extracts of Kalanchoe brasiliensis and Kalanchoe pinnata (Crassulaceae) against Local Effects Induced by Bothrops jararaca Snake Venom
Source: PLoS One. 2017 Feb 16;12(2):e0172598. doi: 10.1371/journal.pone.0172598 (PMC5312879; doi:10.1371/journal.pone.0172598)

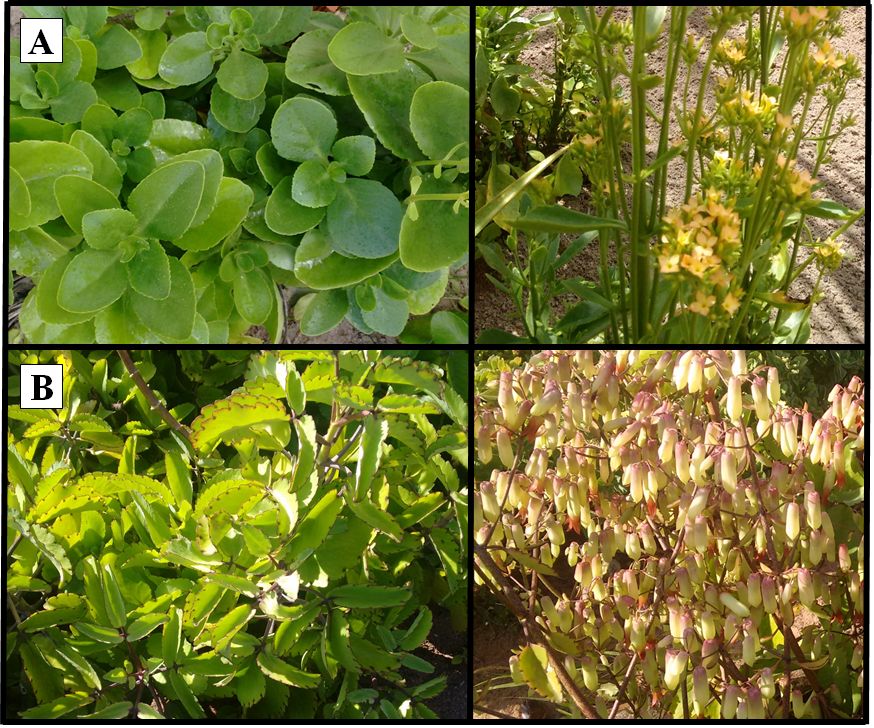

Supplement: S1 Fig — Photography by Júlia Morais Fernandes. doi:10.1371/journal.pone.0168658.s001. (JPG) [file pone.0172598.s001.jpg]
